# Adjuvant atezolizumab in surgically resected NSCLC patients with PD-L1 expression ≥ 50%: real-world data from the Italian ATLAS registry

**DOI:** 10.1093/oncolo/oyaf428

**Published:** 2025-12-24

**Authors:** Francesco Passiglia, Maria Lucia Reale, Giulia Pasello, Giuseppe Viscardi, Ilaria Attili, Francesca Mazzoni, Domenico Galetta, Chiara Catania, Kallopi Andrikou, Alessandro Russo, Tiziana Vavala, Alessandra Bulotta, Lorenzo Calvetti, Anna Maria Carta, Salvatore Grisanti, Sabrina Mariotti, Giulia La Cava, Alessandra Dodi, Vieri Scotti, Teresa Del Giudice, Gabriele Minuti, Elio Gregory Pizzutillo, Rita Chiari, Carminia Maria Della Corte, Carlo Genova, Giuseppe Lo Russo, Daniele Pignataro, Daniele Pozzessere, Elisa Roca, Luca Toschi, Chiara Bennati, Gloria Borra, Anna Bettini, Adolfo Favaretto, Alain Gelibter, Stefania Gori, Fabrizio Tabbò, Maria Pagano, Alberto Pavan, Lorenzo Belluomini, Luca Tondulli, Concetta Sergi, Brigida Stanzione, Umberto Malapelle, Silvia Novello, Emilio Bria

**Affiliations:** Department of Oncology, University of Turin, San Luigi Hospital, Orbassano, TO 10043, Italy; Medical Oncology Unit, “Vito Fazzi” Hospital, Lecce 73100, Italy; DiSCOG University of Padua, Padova 35122, Italy; Oncology Unit 2, IOV Istituto Oncologico Veneto IRCCS, Padova 35128, Italy; UOC Pneumologia Oncologica, AORN Azienda Ospedaliera Dei Colli Monaldi, Napoli 80131, Italy; Thoracic Oncology Division, European Institute of Oncology (IEO), IRCCS, Milan 20141, Italy; Department of Oncology, Careggi University Hospital, Florence 50134, Italy; Medical Thoracic Oncology Unit, IRCCS Istituto Tumori Giovanni Paolo II, Bari 70124, Italy; Medical Oncology Department, Humanitas Gavazzeni, Bergamo 24125, Italy; Medical Oncology Department, IRCCS Istituto Romagnolo per lo Studio dei Tumori (IRST) “Dino Amadori”, Meldola 47014, Italy; Medical Oncology Department, Humanitas Istituto Clinico Catanese, Catania 95045, Italy; Department of Oncology, AOU Città della Salute e della Scienza, SC Oncologia 1U, Torino 10126, Italy; Department of Oncology, IRCCS Ospedale San Raffaele, Milan 20132, Italy; Medical Oncology, San Bortolo Hospital, Vicenza 36100, Italy; SC Oncologia Medica, Ospedale Businco ARNAS G. Brotzu, Cagliari 09121, Italy; Medical Oncology Unit, ASST Spedali Civili di Brescia, Univeristy of Brescia, Brescia 25123, Italy; Medical Oncology, Department of Onco-Hematology, Azienda Ospedaliera Policlinico Tor Vergata, University of Rome Tor Vergata, Rome 00133, Italy; Medical Oncology, Campus Bio-Medico University, Rome 00128, Italy; Medical Oncology Unit, University Hospital of Parma, Parma 43121, Italy; Azienda Ospedaliero-Universitaria Careggi, Florence 50134, Italy; Medical Oncology Unit, AOU Renato Dulbecco, Catanzaro 88100, Italy; Clinical Trials Unit: Phase 1 and Precision Medicine, National Cancer Institute, IRCCS, Regina Elena, Rome 00144, Italy; Niguarda Cancer Center, Grande Ospedale Metropolitano Niguarda, Milan 20162, Italy; UOC Oncologia AST PU—Pesaro e Fano, Pesaro 61121, Italy; Department of Precision Medicine, Università degli studi della Campania “Luigi Vanvitelli”, Naples 80131, Italy; Department of Internal Medicine and Medical Specialties, Academic Medical Oncology Unit, IRCCS Ospedale Policlinico San Martino, University of Genoa, Genoa 16132, Italy; Thoracic Unit, Department of Medical Oncology 1, Fondazione IRCCS Istituto Nazionale dei Tumori di Milano, Milano 20133, Italy; Department of Medical Oncology, Cardinal Massaia Hospital, Asti 14100, Italy; Division of Medical Oncology, S. Stefano Hospital, Azienda USL Toscana Centro, Prato 59100, Italy; Thoracic Oncology—Lung Unit, P. Pederzoli Hospital, Peschiera Del Garda (VR) 37019, Italy; IRCCS Humanitas Clinical and Research Center—Humanitas Cancer Center, Rozzano, Milan 20089, Italy; Oncology Unit, Ospedale Santa Maria delle Croci, Ravenna 48121, Italy; Medical Oncology, Maggiore della Carità Hospital, Novara 28100, Italy; Medical Oncology, ASST Papa Giovanni XXIII, Bergamo 24127, Italy; Department of Medical Oncology, AULSS 2 Marca Trevigiana, Ca’Foncello Hospital, Treviso 31021, Italy; Department of Radiological Sciences, Oncology and Pathology, Sapienza University of Rome, Rome 00185, Italy; Department of Medical Oncology, IRCCS Sacro Cuore Don Calabria Hospital, Negrar di Valpolicella, Verona 37024, Italy; Division of Medical Oncology, ASLCN2 Alba e Bra—Michele e Pietro Ferrero Hospital, Verduno 12060, Italy; Oncologia Medica, IRCCS Arcispedale Maria Nuova Reggio Emilia, Reggio Emilia 42123, Italy; Department of Medical Oncology, AULSS 3 Serenissima, Venezia 30174, Italy; Section of Innovation Biomedicine—Oncology Area, Department of Engineering for Innovation Medicine (DIMI), University of Verona and University and Hospital Trust (AOUI) of Verona, Verona 37126, Italy; Oncologia Medica, Azienda Sanitaria dell’Alto Adige, Bolzano 39100, Italy; Oncology Unit, ARNAS Garibaldi Nesima, Catania 95123, Italy; Dipartimento di Oncologia Medica, Centro di Riferimento Oncologico di Aviano (CRO) IRCCS, Aviano 33081, Italy; Department of Public Health, University Federico II of Naples, Naples 80138, Italy; Department of Oncology, University of Turin, San Luigi Hospital, Orbassano, TO 10043, Italy; Fondazione Policlinico Universitario Agostino Gemelli IRCCS, Università Cattolica del Sacro Cuore, Rome 00168, Italy; Ospedale Isola Tiberina—Gemelli Isola, Rome 00186, Italy

**Keywords:** atezolizumab, adjuvant, PD-L1 ≥ 50%, immunotherapy, non-small cell lung cancer

## Abstract

**Background:**

This study describes clinical characteristics, treatment patterns as well as safety outcomes of NSCLC patients harboring PD-L1 ≥ 50% who received adjuvant atezolizumab within the Italian real-world scenario.

**Methods:**

Patients with surgically resected NSCLC harboring EGFR/ALK wild type disease and PD-L1 TPS ≥ 50%, who received at least one cycle of adjuvant atezolizumab were included. Clinical-pathological and molecular data, safety and efficacy outcomes were collected from the Italian ATLAS real-world registry.

**Results:**

A total of 132 patients were included across 45 Italian centers between July 2022 and August 2024. Lobectomy was performed in 81.1% of cases, with 8.3% pathological stage IIA, 40.2% stage IIB, 43.9% stage IIIA, and 7.6% stage IIIB, according to the eighth TNM staging edition. The median number of atezolizumab cycles was 12.5 (range: 1-20). Treatment related adverse events (TRAEs) during atezolizumab were reported in 44 patients (33.3%), including 11 (8.3%) who experienced multiple TRAEs. Grade ≥ 3 TRAEs were reported in 21 cases (15.9%), leading to treatment discontinuation in 18 (13.6%). The median time to the first onset of TRAEs was 89 days (range: 3-390 days). 15 patients experienced a disease recurrence, including 6 locoregional-only and 9 distant relapses, with a median time since surgery of 13.3 months.

**Conclusion:**

This study showed that the safety profile of adjuvant atezolizumab outside of a clinical trial context was comparable to the IMPower-010 study, highlighting the value of the Italian ATLAS registry as source of real-word evidence to optimize the clinical management of NSCLC patients.

Implications for Practice The safety profile of atezolizumab in surgically resected NSCLC patients treated in the real-world setting was comparable to that reported in the IMPower-010 study. The overall timeline of tocixity onset under adjuvant atezolizumab (median time 90 days) highlights that an accurate clinical monitoring of the patients should be prolonged beyond the first month of therapy. The higher rate of grade 3-4 diarrhea, usually occurring between 60 and 180 days since treatment initiation, suggested that an early detection as well as a proactive management of patients’ symptoms is crucial to optimize tolerability and potentially reducing discontinuation rate in clinical practice.

## Background

Early stage disease accounts for about one third of newly diagnosed non-small cell lung cancer (NSCLC) and this proportion will certainly increase in the near future through the implementation of low-dose computed tomography (LDCT) screening.^1^ Loco-regional treatments, including both surgery and stereotactic body radiotherapy (SBRT), represent a main pillar in the clinical management of these patients, with 5-year survival rate ranging from 90% in stage I to 40% in stage IIIA disease.^2^ This data remind us that lung cancer is a systemic disease since the time of diagnosis and highlight the crucial role of systemic therapies in order to reduce the risk of recurrence and increase the cure rates of NSCLC patients. Following the road marked in the metastatic disease, several studies investigated the efficacy of both targeted therapies and immunotherapy in the neoadjuvant, adjuvant and perioperative setting, revolutioning the treatment paradigm of resectable NSCLC.^3^ In detail the ADAURA^4^ and ALINA^5^ studies marked the advent of targeted therapies, Osimertinib or Alectinib, in the adjuvant treatment of surgically resected NSCLC patients harboring epidermal growth factor receptor (EGFR) activating mutations or anaplastic lymphoma kinase (ALK) fusions, respectively. Six randomized clinical trials^6–11^ investigated neoadjuvant/perioperative chemo-immunotherapy (CheckMate816, CheckMate-77T, AEGEAN, KEYNOTE-671, NeoTorch, RATIONAL-315) in patients with resectable disease, showing a consistent and meaningful benefit, in terms of pathological response and survival outcomes, with 5-year survival rate around 70% in the overall population treated with either neoadjuvant^6^ or perioperative chemo-immunotherapy.^12^ Conversely three randomized clinical trials (IMPower-010, KEYNOTE-091, BR.31) explored the role of consolidation immunotherapy in surgically resected patients who may have received or not adjuvant platinum-chemotherapy, leading to discordant results in terms of disease free survival (DFS).^13–15^ Among these, the IMPower-010 study met its primary endpoint of DFS in surgically resected patients harboring PD-L1 expression ≥ 1%, while a subgroup analysis of the trial based on PD-L1 expression levels, showed that survival benefit was limited to the subgroup with EGFR/ALK wild-type and PD-L1 expression ≥ 50%.^16^^,^^17^ This data supported the regulatory approval of atezolizumab for the adjuvant treatment of patients who underwent surgery followed by platinum-chemotherapy, with some differences between United States and Europe. Indeed the Food and Drugs Administration (FDA) approval was based on the PD-L1 cut-off of 1%, while both the European Medical Agency (EMA) and Italian Drug Agency (AIFA) approval was limited to the subgroup of patients with high PD-L1 expression.

Despite the evidence coming from the randomized IMPower-010 clinical trial, there are currently few data reporting the clinical effectiveness and tolerability of adjuvant atezolizumab in the real-world setting. Here, we reported patients and disease characteristics, treatment patterns as well as safety outcomes of surgically resected NSCLC patients who received adjuvant atezolizumab within the Italian real-world scenario.

## Methods

### Study design and treatment

This is a multicenter, retrospective, observational study conducted on surgically resected NSCLC patients receiving adjuvant atezolizumab in the Italian real-world clinical practice.

Patients were eligible if they were aged ≥18 years; had histologically or cytologically confirmed diagnosis of NSCLC stage I-III (according to the eighth version of the American Joint Committee on Cancer [AJCC]/International Association for the Study of Lung Cancer [IASLC] TNM Staging System); Eastern Cooperative Oncology Group (ECOG) performance status (PS) <3; surgically resected disease; PD-L1 tumor proportion score (TPS) ≥ 50%; without disease recurrence after receiving at least one prior cycle of systemic adjuvant platinum-chemotherapy; received at least one cycle of intravenous (i.v) atezolizumab 1200 mg once every three weeks (q21); participated to the ATLAS real-word registry; signed and dated the ATLAS Informed Consent & privacy Form (ICF) indicating that they understand the purpose and procedures required for the study and are willing to participate in the study and allowing data collection and source data verification in accordance with Italian requirements, if applicable. Clinical, pathological, and molecular data as well as treatment efficacy/tolerability outcomes were retrospectively collected from patients’ medical charts and/or electronic healthcare records across Italian centeres participating to the ATLAS real-word registry and were subsequently archived by using a specific electronic case report form (eCRF) available at the investigators’ sites. Patients who received atezolizumab treatment were ineligible only in case of impossibility to collect the required clinical information. The study was conducted in accordance with the International Conference on Harmonization Guidelines on Good Clinical Practice and the Declaration of Helsinki. The ATLAS protocol was previously approved by the Independent Ethic Committee of the coordinating center at University of Turin (ethics approval number: 0006981) and then at the local Ethic Committees of all the participating centers and all the patients provided a written informed consent before enrollment.

### Objectives and outcomes

The primary objective of this study is to assess the safety and tolerability profile of adjuvant atezolizumab in surgically resected NSCLC patients with PD-L1 TPS ≥ 50% in order to provide a reliable picture of treatment-tolerability in the real-world clinical setting.

The primary outcome of the study includes the incidence of treatment-related adverse events (TRAEs) under atezolizumab therapy, according to the Common Terminology Criteria (CTCAE version 5.0).

The secondary objectives of this study are to describe clinical, pathological, and molecular characteristics of included patients, treatment patterns, and their potential correlation with atezolizumab tolerability in surgically resected NSCLC patients with PD-L1 TPS ≥ 50%.

The secondary outcomes of the study include any differences of atezolizumab safety outcomes across specific patients’ subgroups selected according to the following characteristics: smoking status, ECOG PS, age, tumor type, disease stage, adjuvant platinum chemotherapy, adjuvant radiotherapy, PD-L1 TPS, oncogenic driver alterations.

### Statistical analysis

The number and percentage of participants receiving atezolizumab therapy as well as their clinical, pathological, molecular characteristics, and administered therapies have been summarized either by descriptive statistics or categorical tables. Descriptive analysis has been performed, including means, standard deviations, medians, quartiles, and absolute/relative frequencies (with their respective two-sided 95% confidence interval [CIs] limits, where relevant), according to the specific variables. The Mann Whitney test was used for intergroup comparisons of two independent samples while Fisher’s test was used for categorical values, by using a *P* value < .05 as threshold for statistical significance. Radiological evaluation of treatment efficacy by CT-scan was performed every 3-6 months according to the local practice. TRAEs have been reported and graded in severity according to the NCTCAE version 5.0. The number of months of treatment have been investigated by summarizing the number of months from the first dose of study drug to the last dose of study drug. The number of patients with at least one dose reduction or interruption has been summarized with frequencies and percentages reported. The statistical analysis has been performed by using SPSS Statistics software version 20 (IBM, Armonk, New York, USA).

## Results

### Patients’ characteristics

From July 2022 to August 2024, 132 patients who underwent surgical resection and received atezolizumab in the adjuvant setting across 43 Italian centers were considered eligible and included in the analysis. Patients’ and disease characteristics are summarized in Table 1. The median age at diagnosis was 65 years (range: 35-80). Most patients were male (*n* = 86, 65.2%), current/former smokers (*n* = 120, 90.9%), with an Eastern Cooperative Oncology Group (ECOG) performance status (PS) of 0 (61.4%) and 1 (37.9%).

**Table 1.** Characteristics of the patients.

Characteristic	*N* (%)
**Age**	
** Median—years**	65 (range 35-80)
** <65 years**	61 (46.2)
** ≥65 years**	71 (53.8)
**Sex**	
** Female**	46 (34.8)
** Male**	86 (65.2)
**ECOG performance status**	
** 0**	81 (61.4)
** 1**	50 (37.9)
** 2**	1 (0.8)
**Relevant comorbidities**	
** No**	118 (89.4)
** Yes**	14 (11.6)
** Chronic obstructive pulmonary disease**	9 (6.8)
** Autoimmune disease**	2 (1.5)
** Cardiovascular disease**	3 (2.3)
**Smoking status**	
** Never smoked**	7 (5.3)
** Former smoker**	43 (32.6)
** Current smoker**	77 (58.3)
** Unknown**	5 (3.8)
**Pathological stage**	
** IIA**	11 (8.3)
** IIB**	53 (40.2)
** IIIA**	58 (43.9)
** IIIB**	10 (7.6)
**Loco-regional lymphnodes**	
** N0**	31 (23.5)
** N1**	55 (41.7)
** N2**	46 (34.8)
**Histologic subtype**	
** Squamous**	40 (30.3)
** Adenocarcinoma**	82 (62.1)
** Other**	10 (7.6)
**Surgery**	
** Lobectomy**	107 (81.1)
** Bilobectomy**	4 (3.0)
** Pneumonectomy**	9 (6.8)
** Wedge resection**	4 (3.0)
** Unknown**	8 (6.1)
**Residual tumor**	
** R0**	129 (97.7)
** R1**	3 (2.3)
**PD-L1 TPS**	
** 50%-89%**	95 (72.0)
** 90%-100%**	29 (22.0)
** Not specified**	8 (6.1)

PD-L1 TPS, Programmed death ligand-1 Tumor Proportion Score.

The most common histological subtype was adenocarcinoma (62.1%), with 29 cases (22.0%) harboring a PD-L1 expression higher than 90%. Molecular analyses were mainly performed using either next generation sequencing (NGS) (84 patients, 63.6%) or RT-PCR (21 patients, 15.9%). All patients were *EGFR* and *ALK* wild-type. Other molecular alterations were detected in 63 cases (47.7%), with *KRAS* G12C and non-G12C mutations identified in 21 (15.9%) and 24 (18.2%) patients, respectively. A detailed description of the molecular alterations identified in the study population is reported in the **Table S1**.

The therapeutic strategy for most of the cases (83.3%) was defined within a multidisciplinary team discussion. Lobectomy was performed in 107 patients (81.1%), followed by pneumonectomy (*n* = 9, 6.8%), bilobectomy (*n* = 4, 3.0%), and wedge resection (*n* = 4, 3.0%), while the surgical approach was not reported in 8 (6.1%) of cases. A total of 129 patients (97.7%) underwent complete resection with negative margins. Based on the eighth TNM staging edition, 11 (8.3%) patients had pathological stage IIA, 53 (40.2%) stage IIB, 58 (43.9%) stage IIIA, and 10 (7.6%) stage IIIB. All the patients received adjuvant chemotherapy. The most common regimens were either cisplatin plus vinorelbine (received by 77 patients, 58.3%) or cisplatin plus gemcitabine (received by 32 patients, 24.2%). Only 3.8% (*n* = 5) of the patients received other cisplatin-based regimens, while 13.6% (*n* = 18) of them a carboplatin-based combination (**Table S2**). The median time from surgery to the adjuvant treatment initiation was 59 days (range 13-270). The median number of adjuvant chemotherapy cycles administered was four (range: 1-4), with 112 patients (84.8%) receiving all of them. Two patients (1.5%) with pathological (p)N2 disease underwent adjuvant radiotherapy. At the data cutoff (December 22, 2024), the median duration of follow-up from adjuvant atezolizumab initiation was 15.7 months. Overall 72 out of 132 patients (54.4%) were still on treatment with adjuvant atezolizumab. Among the others, the median number of atezolizumab cycles was 12.5 (range: 1-20), with 34 patients completing the full course of 1-year immunotherapy consolidation.

### Safety analysis

The AEs considered by the investigator to be causally related to the adjuvant treatment occurred in 88 (66.7%) patients, with the majority associated with adjuvant chemotherapy (54 patients, 41.0%). The incidence of grade 3 or 4 chemotherapy-related AEs was 12.1% (*n* = 16). The most common grade 3 or 4 chemotherapy-related AEs were neutropenia (3.8%), thrombocytopenia (1.5%) and increased creatinine levels (1.5%) (**Table S3**). TRAEs of any grade leading to discontinuation of adjuvant chemotherapy occurred in 20 (15.2%) patients.

**Figure 1.**
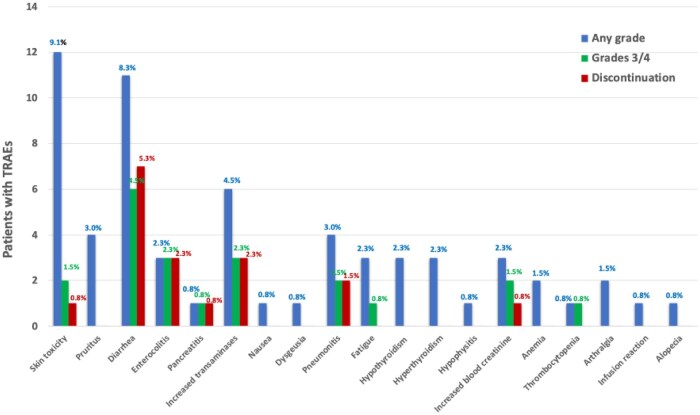
Incidence of TRAEs and discontinuation rates with adjuvant atezolizumab in NSCLC patients.

Adverse events considered causally related to adjuvant atezolizumab were reported in 44 patients (33.3%), including 11 patients (8.3%) who experienced multiple TRAEs (Table 2). Of these, 10 patients (23%) had previously experienced chemo-related toxicities, leading to a definitive chemotherapy discontinuation in 4 cases (9%). The most common immune-related AEs of any grade were skin rash (*n* = 12, 9.1%), diarrhea (*n* = 11, 8.3%), and elevated transaminases (*n* = 6, 4.5%). Grade ≥ 3 TRAEs were reported in 21 cases (15.9%), with the most common being diarrhea (*n* = 6, 4.5%), elevated transaminases (*n* = 3, 2.3%), and enterocolitis (*n* = 3, 2.3%). TRAEs leading to treatment discontinuation were reported in 18 patients (13.6%). The most common were diarrhea (*n* = 7, 5.3%), increased transaminases (*n* = 3, 2.3%), and enterocolitis (*n* = 3, 2.3%) (Figure 1). TRAEs leading to temporary dose interruption were reported in four patients (3.0%), including skin toxicity (*n* = 2, 1.5%), hypophysitis (*n* = 1, 0.8%), and pneumonitis (*n* = 1, 0.8%).

**Table 2.** Treatment-Related Adverse Events (TRAEs) with adjuvant atezolizumab.

Treatment-Related Adverse Events (TRAEs) with adjuvant atezolizumab^a^	*N* = 132 (%)
**Any grade TRAEs^a^**	
** Grade 1**	19 (14.4)
** Grade 2**	18 (13.6)
** Grade 3**	18 (13.6)
** Grade 4**	3 (2.3)
**Grade 3 TRAEs^a^**	
** Diarrhea**	6 (4.5)
** Increased transaminases**	3 (2.3)
** Pancreatitis**	1 (0.8)
** Pneumonitis**	2 (1.5)
** Fatigue**	1 (0.8)
** Increased blood creatinine**	2 (1.5)
** Thrombocytopenia**	1 (0.8)
** Skin toxicity**	2 (1.5)
**Grade 4 TRAEs^a^**	
** Enterocolitis**	3 (2.3)
**TRAE leading to discontinuation of therapy**	
** Skin toxicity**	1 (0.8)
** Diarrhea**	7 (5.3)
** Enterocolitis**	3 (2.3)
** Pancreatitis**	1 (0.8)
** Increased transaminases**	3 (2.3)
** Pneumonitis**	2 (1.5)
** Increased blood creatinine**	1 (0.8)

a Some patients reported multiple TRAEs.

**Figure 2.**
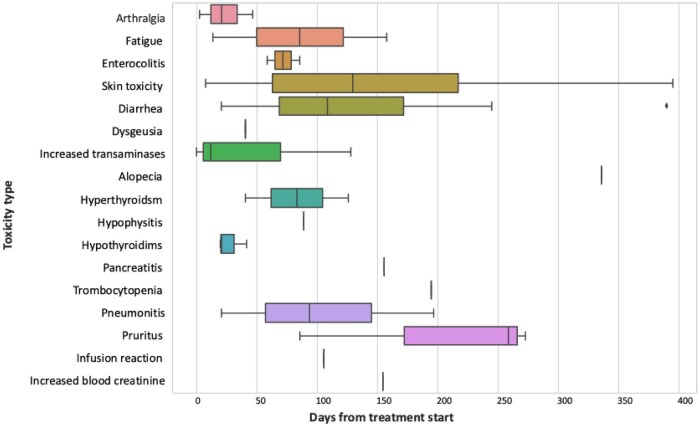
Time-to-onset distribution of TRAEs with adjuvant atezolizumab in NSCLC patients.

The median time to the first onset of atezolizumab-related AEs was 89 days (range: 3-390 days), with only 25% of patients experiencing TRAEs within one month since treatment initiation. A detailed timeline for the different toxicities was reported in Figure 2. Overall, Immune-mediated AEs requiring systemic corticosteroid treatment occurred in 21 patients (15.9%), most commonly for diarrhea (4.5%). Immunosuppressive therapy was required in two cases (1.5%) of enterocolitis. Complete resolution of immune-related AEs was observed in 26 patients (19.7%).

The frequency of both any grade and severe TRAEs was generally similar across the main patients’ subgroups, including sex, age, smoking status, tumor PD-L1 expression level, surgical approaches (lobectomy vs pneumonectomy). A significantly higher incidence of grade ≥ 3 TRAEs was observed in patients with ECOG-PS ≥ 1 (29.4% vs. 7.4%, *P*:.001) and in patients who did not experience toxicities under adjuvant chemotherapy (24.4% vs. 3.7%, *P*: .001) (Table 3).

**Table 3.** TRAEs differences with adjuvant atezolizumab across main patients’ subgroups.

TRAEs with adjuvant atezolizumab	Male (*n* = 86)	Female (*n* = 46)	*P* value
**Any TRAE**	28 (32.6%)	16 (34.8%)	.85
**TRAE grade ≥ 3^a^**	14 (16.3%)	7 (15.2%)	1
**TRAE leading to discontinuation**	12 (14.0%)	6 (13.0%)	1
	**<65 years (*n* = 61)**	**≥65 years (*n* = 71)**	
**Any TRAE**	22 (36.1%)	22 (31.0%)	.58
**TRAE grade ≥ 3^a^**	10 (16.4%)	11 (15.5%)	1
**TRAE leading to discontinuation**	9 (14.8%)	9 (12.7%)	.80
	**ECOG PS 0 (*n* = 81)**	**ECOG PS ≥ 1 (*n* = 51)**	
**Any TRAE**	26 (32.1%)	18 (35.3%)	.71
**TRAE grade ≥ 3^a^**	6 (7.4%)	15 (29.4%)	.001^b^
**TRAE leading to discontinuation**	7 (8.6%)	11 (21.6%)	.04^b^
	**Current smokers (*n* = 77)**	**Never/former smokers (*n* = 50)**	
**Any TRAE**	27 (35.1%)	17 (34.0%)	1
**TRAE grade ≥ 3^a^**	12 (15.6%)	9 (18.0%)	.81
**TRAE leading to discontinuation**	11 (14.3%)	7 (14.0%)	1
	**PD-L1 50-89% (*n* = 95)**	**PD-L1 90-100% (*n* = 29)**	
**Any TRAE**	32 (33.7%)	12 (41.4%)	.51
**TRAE grade ≥ 3^a^**	14 (14.7%)	7 (24.1%)	.26
**TRAE leading to discontinuation**	12 (12.6%)	6 (20.1%)	.36
	**Pneumonectomy (*n* = 9)**	**No pneumonectomy (*n* = 115)**	
**Any TRAE**	3 (33.3%)	41 (35.7%)	1
**TRAE grade ≥ 3^a^**	2 (22.2%)	19 (16.5%)	.65
**TRAE leading to discontinuation**	1 (11.1%)	17 (14.8%)	1
	**No chemo toxicity (*n* = 78)**	**Previous chemo toxicity (*n* = 54)**	
**Any TRAE**	34 (43.6%)	10 (18.5%)	.003^b^
**TRAE grade ≥ 3^a^**	19 (24.4%)	2 (3.7%)	.001^b^
**TRAE leading to discontinuation**	14 (17.9%)	4 (7.4%)	.12

TRAEs: treatment-related adverse events.

a Some patients reported multiple AEs.

b Statistically significant.

### Efficacy analysis

With a median follow-up of 15.7 months from the start of adjuvant atezolizumab, 15 patients (11.3%) experienced a disease recurrence with a median time to relapse from surgery of 13.3 months. One third of these patients had KRAS mutations (3 patients KRAS G12C mutations and two non-G12C KRAS mutations). In nine cases (60%), PD-L1 expression ranged between 50% and 80%. Most of these patients (66.7%) underwent lobectomy. Most had pathological stage III disease (40% stage IIIA and 20% stage IIIB) and received cisplatin-based adjuvant chemotherapy (93.3%), with 66.7% completing all four cycles. The median number of adjuvant atezolizumab cycles was 11 (range 3-16). Eight patients (53.3%) progressed while receiving adjuvant atezolizumab (median time to relapse from surgery of 12.2 months). Three patients (20.0%) had discontinued adjuvant immunotherapy due to toxicities (median time to relapse from surgery of 16.1 months). Four patients (26.7%) experienced disease progression after completing adjuvant atezolizumab with a median time to relapse from surgery of 16.2 months.

Specifically, locoregional-only recurrence was observed in six out of 15 patients (40%) while nine of them (60%) developed distant relapse, including pleura (2, 13%), hepatic (2, 13%), soft tissue (1, 6.5%), bone (1, 6.5%) and central nervous system (3, 20%) involvement. Local therapy (radiotherapy or surgical resection) was performed in four patients (27%), concurrent chemoradiation in two patients (13%) and systemic treatment in four patients (27%). Two patients received a platinum-based chemotherapy and the others two a mono-chemotherapy after a median interval of 8.5 and 5.2 months, respectively, from the completion of adjuvant chemotherapy. Information on subsequent treatments was lacking for five patients. Two deaths (1.5%) occurred among patients with disease recurrence, while all other patients were alive at the time of data analysis.

## Discussion

To the best of our knowledge this is the first real-world clinical study describing the safety profile of adjuvant atezolizumab in surgically resected NSCLC patients. The clinical characteristics of the patients were quite similar to those reported in the PD-L1 ≥ 50% population included in the IMPower-010 randomized trial.^15–17^ However a higher number of females and elderly (>65 years/old) patients were included in this analysis, as well as a small subgroup with pathological stage IIIB disease who received adjuvant atezolizumab in the real-world. Differently from the IMPower-010 study, only EGFR and ALK wild-type patients can receive adjuvant atezolizumab in Italy, in accordance to both the AIFA approval indications as well as the European Society of Medical Oncology (ESMO) and Italian Association of Medical Oncology (AIOM) lung cancer guidelines. However the vast majority of patients underwent NGS-based molecular profiling on surgical samples, showing molecular alterations *(KRAS*, *BRAF*, *TP53*, *PI3KCA*) considered responsive to immunotherapy. Looking at the surgical approach, it’s interesting to note a significant lower rate of pneumonectomy in this real-world analysis, even if our data, in line with the IMPower-010 study^18^ showed no differences in the tolerability profile of adjuvant atezolizumab regardless of the surgical approach. The administration of 4 cycles of cisplatin-based combinations in the vast majority of cases and the initiation of adjuvant chemotherapy within 2 months since surgery, suggest a high adherence of Italian medical oncologists to the clinical practice guidelines and support the feasibility of this treatment in a real-world population. However, the lack of patients with ECOG-PS 2 confirms that an accurate clinical selection of best candidates for adjuvant regimens remains crucial. The results of this analysis showed that the safety profile of atezolizumab in surgically resected NSCLC patients treated outside of a clinical trial context was comparable to that reported in the IMPower-010 study, with a similar rate of grade ≥ 3 TRAEs as well as treatment withdrawal, confirming an optimal tolerability also in a real-world population. Conversely the lower rate of overall TRAEs could be related to an underestimation and/or undereporting of low grade toxicities, suggesting a not uniform approach for safety data collection between clinical trial and real-word analysis. The overall timeline of tocixity onset under adjuvant atezolizumab highlights that an accurate clinical monitoring of the patients should be prolonged beyond the first months of therapy. Differently from the IMPower-010 trial, our anlysis showed a higher rate of severe diarrhea, usually occurring between 60 and 180 days since treatment initiation, suggesting that an early detection as well as a proactive management of patients’ symptoms is crucial to optimize the tolerability profile of atezolizumab and potentially reducing the discontinuation rate of such curative treatment in clinical practice. Our study also showed a significantly higher incidence of severe TRAEs as well as treatment discontinuation rate in patients with ECOG-PS 1, confirming once again the crucial role of an accurate clinical selection of best candidates to adjuvant treatment. Differently from what expected, previous toxicity occurring during adjuvant chemotherapy were not associated with a higher risk of immune-related toxicity, suggesting that a bad tolerability of platinum-chemo should not be used as a criteria to exclude patients from atezolizumab consolidation. The limited follow-up and the low rate of disease relapse at the time of data analysis do not allow to speculate about the overall treatment compliance as well as potential efficacy of adjuvant atezolizumab in a real-world population as compared to a clinical trial setting. Even if both patterns of disease recurrence and subsequent treatments seem to be in line with those reported in the randomized study, additional data from a larger population, at longer follow-up, is warranted to elucidate the real-world management practices and efficacy outcomes in this emerging clinical context.

Although this study is limited by retrospective design and limited follow-up, it likely represents the largest real-word descriptive analysis of the tolerability profile of adjuvant atezolizumab in EGFR/ALK wild-type and PD-L1 ≥ 50%, surgically resected NSCLC patients, highlighting the role of the multicenter italian ATLAS registry,^14^ as source of real-word evidence to optimize the clinical management of resectable NSCLC patients.

## Author contributions

Francesco Passiglia (Conceptualization, Data curation, Formal analysis, Writing—original draft), Maria Lucia Reale (Data curation, Formal analysis, Writing—original draft), Giulia Pasello (Data curation, Validation, Visualization), Giuseppe Viscardi (Data curation, Validation, Visualization), Ilaria Attili (Data curation, Validation, Visualization), Francesca Mazzoni (Data curation, Validation, Visualization), Domenico Galetta (Data curation, Validation, Visualization), Chiara Catania (Data curation, Validation, Visualization), Kallopi Andrikou (Data curation, Validation, Visualization), Alessandro Russo (Data curation, Validation, Visualization), Tiziana Vavala’(Data curation, Validation, Visualization), Alessandra Bulotta (Data curation, Validation, Visualization), Lorenzo Calvetti (Data curation, Validation, Visualization), Anna Maria Carta (Data curation, Validation, Visualization), Salvatore Grisanti (Data curation, Validation, Visualization), Sabrina Mariotti (Data curation, Validation, Visualization), Giulia La Cava (Data curation, Validation, Visualization), Alessandra Dodi (Data curation, Validation, Visualization), Vieri Scotti (Data curation, Validation, Visualization), Teresa Del Giudice (Data curation, Validation, Visualization), Gabriele Minuti (Data curation, Validation, Visualization), Elio Gregory Pizzutillo (Data curation, Validation, Visualization), Rita Chiari (Data curation, Validation, Visualization), Carminia Maria Della Corte (Data curation, Validation, Visualization), Carlo Genova (Data curation, Validation, Visualization), Giuseppe Lo Russo (Data curation, Validation, Visualization), Daniele Pignataro (Data curation, Validation, Visualization), Daniele Pozzessere (Data curation, Validation, Visualization), Elisa Roca (Data curation, Validation, Visualization), Luca Toschi (Data curation, Validation, Visualization), Chiara Bennati (Data curation, Validation, Visualization), Gloria Borra (Data curation, Validation, Visualization), Anna Bettini (Data curation, Validation, Visualization), Adolfo Favaretto (Data curation, Validation, Visualization), Alain J Gelibter (Data curation, Validation, Visualization), Stefania Gori (Data curation, Validation, Visualization), Fabrizio Tabbò (Data curation, Validation, Visualization), Maria Pagano (Data curation, Validation, Visualization), Alberto Pavan (Data curation, Validation, Visualization), Lorenzo Belluomini (Data curation, Validation, Visualization), Luca Tondulli (Data curation, Validation, Visualization), Concetta Sergi (Data curation, Validation, Visualization), Brigida Stanzione (Data curation, Validation, Visualization), Umberto Malapelle (Data curation, Validation, Visualization), and Silvia Novello (Conceptualization, Supervision, Validation, Visualization), Emilio Bria (Conceptualization, Data curation, Supervision, Validation, Visualization)

## Supplementary Material

oyaf428_Supplementary_Data

## Data Availability

Additional data can be found in the Supplementary data of this article. Queries for any data not included in the article or its Supplementary files may be directed to the corresponding author.
