# Supplementary material for: Adjuvant atezolizumab in surgically resected NSCLC patients with PD-L1 expression ≥ 50%: real-world data from the Italian ATLAS registry
Source: Oncologist. 2025 Dec 24;31(2):oyaf428. doi: 10.1093/oncolo/oyaf428 (PMC12854775; doi:10.1093/oncolo/oyaf428)
Supplement: oyaf428_Supplementary_Data [file oyaf428_supplementary_data.zip › Supplemenatry Table 2..docx]

| **Adjuvant Chemotherapy schemes** | **N (%)** |
| --- | --- |
| Carboplatin-pemetrexed | 4 (3.0) |
| Cisplatin-pemetrexed | 3 (2.3) |
| Cisplatin-etoposide | 1 (0.8) |
| Cisplatin-vinorelbine | 77 (58.3) |
| Cisplatin-gemcitabine | 32 (24.2) |
| Carboplatin-gemcitabine | 7 (5.3) |
| Carboplatin-vinorelbine | 7 (5.3) |
| Cisplatin-docetaxel | 1 (0.8) |

**Supplementary Table 2.Adjuvant chemotherapy schemes (N=132)**
