# Supplementary material for: Adjuvant atezolizumab in surgically resected NSCLC patients with PD-L1 expression ≥ 50%: real-world data from the Italian ATLAS registry
Source: Oncologist. 2025 Dec 24;31(2):oyaf428. doi: 10.1093/oncolo/oyaf428 (PMC12854775; doi:10.1093/oncolo/oyaf428)
Supplement: oyaf428_Supplementary_Data [file oyaf428_supplementary_data.zip › Supplementary Table 1..docx]

**Supplementary Table 1. Molecular features of the patients at baseline (N=132)**

| **Molecular features** | N (%) |
| --- | --- |
| ***EGFR* status** | |
| Wilde type | 132 (100) |
| ***ALK* status** | |
| Wilde type | 132 (100) |
| ***ROS1* status** | |
| Wilde type | 92 (69.7) |
| Unknown | 40 (30.3) |
| ***RET* status** | |
| Wilde type | 85 (64.4) |
| Unknown | 47 (35.6) |
| ***MET* status** | |
| Wilde type | 84 (63.6) |
| Unknown | 48 (36.3) |
| ***HER2* status** | |
| *HER2* mutation | 1 (0.8) |
| Wilde type | 81 (61.4) |
| Unknown | 50 (37.9) |
| ***KRAS* status** | |
| *KRAS* G12C | 21 (15.9) |
| *KRAS* non-G12C | 24 (18.2) |
| Wild type | 46 (34.8) |
| Unknown | 41 (31.1) |
| ***BRAF* status** | |
| *BRAF* V600E | 1 (0.8) |
| *BRAF* non-V600 | 4 (3.0) |
| Wilde Type | 86 (65.2) |
| Unkown | 41 (31.1) |
| ***NTRK* status** | |
| Wilde Type | 85 (64.4) |
| Unkown | 47 (35.6) |
| **Other alterations** |  |
| *PI3KCA* mutation | 4 (3.0) |
| *TP53* mutation | 10 (7.6) |
| *PTEN* mutation | 1 (0.8) |
| *STK11* mutation | 1 (0.8) |
