# Supplementary material for: Adjuvant atezolizumab in surgically resected NSCLC patients with PD-L1 expression ≥ 50%: real-world data from the Italian ATLAS registry
Source: Oncologist. 2025 Dec 24;31(2):oyaf428. doi: 10.1093/oncolo/oyaf428 (PMC12854775; doi:10.1093/oncolo/oyaf428)
Supplement: oyaf428_Supplementary_Data [file oyaf428_supplementary_data.zip › Supplementary Table 3..docx]

| **TRAEs^a^** | **Any Grade n (%)** | **Grade > 3 n (%)** |
| --- | --- | --- |
| All | 54 (40.9) | 16 (12.1%) |
| Peripheral sensory neuropathy | 3 (2.3) | 1 (0.8) |
| Nausea | 8 (6.1) | 1 (0.8) |
| Fatigue | 7 (5.3) | 1 (0.8) |
| Creatinine increase | 6 (4.5) | 2 (1.5) |
| Neutropenia | 9 (6.8) | 5 (3.8) |
| Anemia | 4 (3.0) | 1 (0.8) |
| Thrombocytopenia | 5 (3.8) | 2 (1.5) |
| Cardiotoxicity | 2 (1.5) | 1 (0.8) |
| Dysgeusia | 3 (2.3) | 0 (0) |
| Alopecia | 2 (1.5) | 0 (0) |
| Constipation | 2 (1.5) | 0 (0) |
| Increased transaminases | 1 (0.8) | 1 (0.8) |
| Colitis | 1 (0.8) | 1 (0.8) |
| Ototoxicity | 1 (0.8) | 0 (0) |

^a^Some patients reported multiple TRAEs.
